# Post-Discharge Mortality in Children with Severe Malnutrition and Pneumonia in Bangladesh

**DOI:** 10.1371/journal.pone.0107663

**Published:** 2014-09-16

**Authors:** Mohammod Jobayer Chisti, Stephen M. Graham, Trevor Duke, Tahmeed Ahmed, Abu Syed Golam Faruque, Hasan Ashraf, Pradip Kumar Bardhan, Abu S. M. S. B. Shahid, K. M. Shahunja, Mohammed Abdus Salam

**Affiliations:** 1 International Centre for Diarrhoeal Disease Research, Bangladesh, Dhaka, Bangladesh; 2 Centre for International Child Health, The University of Melbourne Department of Paediatrics and Murdoch Childrens Research Institute, Royal Children’s Hospital, Melbourne, Australia; 3 International Union Against Tuberculosis and Lung Disease, Paris, France; Fondazione IRCCS Ca’ Granda Ospedale Maggiore Policlinico, Università degli Studi di Milano, Italy

## Abstract

**Background:**

Post-discharge mortality among children with severe illness in resource-limited settings is under-recognized and there are limited data. We evaluated post-discharge mortality in a recently reported cohort of children with severe malnutrition and pneumonia, and identified characteristics associated with an increased risk of death.

**Methods:**

Young children (<5 years of age) with severe malnutrition (WHO criteria) and radiographic pneumonia on admission to Dhaka Hospital of icddr,b over a 15-month period were managed according to standard protocols. Those discharged were followed-up and survival status at 12 weeks post-discharge was determined. Verbal autopsy was requested from families of those that died.

**Results:**

Of 405 children hospitalized with severe malnutrition and pneumonia, 369 (median age, 10 months) were discharged alive with a follow-up plan. Of these, 32 (8.7%) died in the community within 3 months of discharge: median 22 (IQR 9–35) days from discharge to death. Most deaths were reportedly associated with acute onset of new respiratory or gastrointestinal symptoms. Those that died following discharge were significantly younger (median 6 [IQR 3,12] months) and more severely malnourished, on admission and on discharge, than those that survived. Bivariate analysis found that severe wasting on admission (OR 3.64, 95% CI 1.66–7.97) and age <12 months (OR 2.54, 95% CI 1.1–8.8) were significantly associated with post-discharge death. Of those that died in the community, none had attended a scheduled follow-up and care-seeking from a traditional healer was more common (p<0.001) compared to those who survived.

**Conclusion and Significance:**

Post-discharge mortality was common in Bangladeshi children following inpatient care for severe malnutrition and pneumonia. The underlying contributing factors require a better understanding to inform the potential of interventions that could improve survival.

## Introduction

Malnutrition is recognized as a major underlying risk factor for death in children with common infections causing global child mortality such as pneumonia, diarrhea and sepsis [1], [2]. Furthermore, these infectious diseases further increase the high risk of mortality that is reported in children with severe malnutrition managed as inpatients in resource-poor settings of Africa and Asia [3]–[7]. Malnutrition is also a consistent risk factor for post-discharge death in common childhood illnesses in high-mortality settings, including studies from Bangladesh of children hospitalized with diarrhea [8]–[10]. However, there are limited data available from studies that have followed severely malnourished children following discharge from inpatient facilities. Currently, these data are all from settings in sub-Saharan Africa [5], [11], [12] and previous studies have not reported post-discharge death in children with severe malnutrition and pneumonia.

Bangladesh has high rates of childhood malnutrition. A UNICEF report published in 2013 reported that 36% of Bangladeshi children had moderate or severe wasting and that 41% had moderate or severe stunting in the period 2007 to 2011 [13]. We recently undertook a study to determine bacterial and mycobacterial aetiology of pneumonia in children with severe malnutrition admitted to the Dhaka Hospital of the International Centre for Diarrhoeal Disease Research, Bangladesh (icddr’b) in Dhaka, Bangladesh [14]. The aim of this analysis was to report inpatient and early post-discharge mortality in this same cohort of children with severe malnutrition and radiological pneumonia, and to identify characteristics associated with an increased risk of death following discharge.

## Materials and Methods

### Ethics statement

The study (protocol number: PR-10067) was approved by the Research Review Committee (RRC) and the Ethical Review Committee (ERC) of icddr,b. Written informed consent was obtained from parents or attending guardians of all the participating children.

### Study setting and design

This was a prospective study cohort study that included young children (0–59 months of age) with severe malnutrition, respiratory symptoms and radiological pneumonia as previously described in detail [14]. In brief, eligible children admitted to the Dhaka Hospital of icddr,b between April 2011 and June 2012 were enrolled following informed consent. Demographic, contact details (phone and address) and clinical data were collected prospectively on standardized data collection proforma. Radiographs were interpreted independently by a radiologist and a study pediatrician (MJC). Radiological pneumonia was defined as the presence of end-point consolidation or other (non-end-point) infiltrate in lungs according to the WHO radiological classification of pneumonia [15]. Severe malnutrition was defined in children as severe wasting [z score for weight for height <−3 of the median of the WHO anthropometry] or severe underweight [z score for weight for age <−4 of the median of the WHO anthropometry], or the presence of nutritional edema. Clinical management was according to standardized guidelines with regards to antibiotics, detection and management of hypoxemia and hypoglycemia, fluid management and nutritional support [14], [16].

Once children were considered to have shown clinical improvement for pneumonia and stabilized, they were transferred to the nutritional rehabilitation unit (NRU) for ongoing care before hospital discharge and were managed according to standard guidelines [6]. During the NRU stay, mothers are taught to prepare high energy food from locally available and culturally acceptable foodstuffs, and advised to provide such food once discharged. The decision to discharge is normally based on the criteria used by the NRU staff, i.e. weight gain of >15% of admission weight (after resolution of edema in those with nutritional edema). However, many children are discharged before these criteria are reached and are discharged “on risk bond”. The main reason is request for early discharge by the mother because of pressure from the child’s father or grandparents for the mother (and child) to return home once the child is thought to be no longer acutely ill. No additional nutritional supplements such as Ready-to-Use Therapeutic Food (RUTF) are provided on discharge.

The primary outcome for analysis was survival status at 3 months post-discharge. Secondary outcomes included the type and duration of new symptoms associated with those that died as reported by verbal autopsy and the proportion of those discharged that were lost-to-follow-up.

### Follow-up procedure

All of the enrolled children that survived hospitalization were requested to attend follow-up for 3 months post-discharge as per routine follow-up by NRU. This included children that were discharged either as left against medical advice or those discharged “on risk bond” from the NRU. Follow-up was planned weekly for the first 2 weeks following discharge and monthly thereafter until the following criteria are met: weight for length ≥−1 z score and/or weight for age ≥−2 z score. Routine follow-up included measurement of nutritional parameters and vital signs, as well as assessment for and management of any intercurrent illness. Neither additional nutritional supplements such as RUTF nor support for transport costs were provided at follow-up visits.

There were 86 children discharged that had been diagnosed with tuberculosis, of which 27 were microbiologically confirmed [14]. It was planned that all children discharged receiving anti-tuberculosis treatment were followed until completion of the course i.e. for 6 months.

For this study, all care-givers were provided instructions verbally in local language regarding follow-up. They were requested to return to the hospital at 12 weeks following discharge but advised that if their children developed any symptoms or signs of illness at any time prior to this, then they should either return to the hospital or consult with a study physician by mobile phone at any time. The phone numbers of the study physicians were provided during discharge and all caregivers had access to mobile phones. No funding was provided for potential phone call costs. If any of the caregivers did not attend the 12-week post-discharge follow-up, the study staff attempted to contact them via mobile phone. If any of the care-givers could not be contacted by mobile phone or still did not attend the follow-up after contact by mobile phone, then research assistants visited their home to ensure follow-up. Study participants were defined as “lost to follow-up” if research assistants visited the given address on at least two separate occasions and failed to identify them.

Verbal autopsy was requested when possible for those children that were identified as having died during the follow-up period. Verbal autopsy procedure followed the WHO standard questionnaire [17]. Information obtained included the presence and duration of symptoms prior to death as well as treatment sought. Interviews were undertaken by the study physicians at icddr’b for the majority that were willing to attend, otherwise the interview was performed by mobile phone (n = 4).

### Data analysis

All data were entered into SPSS for Windows (version 15.0; SPSS Inc, Chicago) and Epi-Info (version 6.0, USD, Stone Mountain, GA). Follow-up was planned prospectively and ethically approved. However, the primary objective of this study that determined sample size was to determine prevalence of sepsis and tuberculosis, as previously reported [14]. We used this sample of patients to determine characteristics associated with post-discharge death. Differences in proportions were compared by the Chi-square test. Differences of means were compared by Student’s t-test for normally distributed data and Mann-Whitney test was used for comparison of data that were not normally distributed. Strength of association was determined by calculating odds ratio (OR) and their 95% confidence intervals (CIs). Two pragmatic categorical variables (age and severity of malnutrition on admission) were identified based on previous literature [8] and univariate analysis of the dataset for bivariate analysis using logistic regression.

## Results

**Figure 1:**
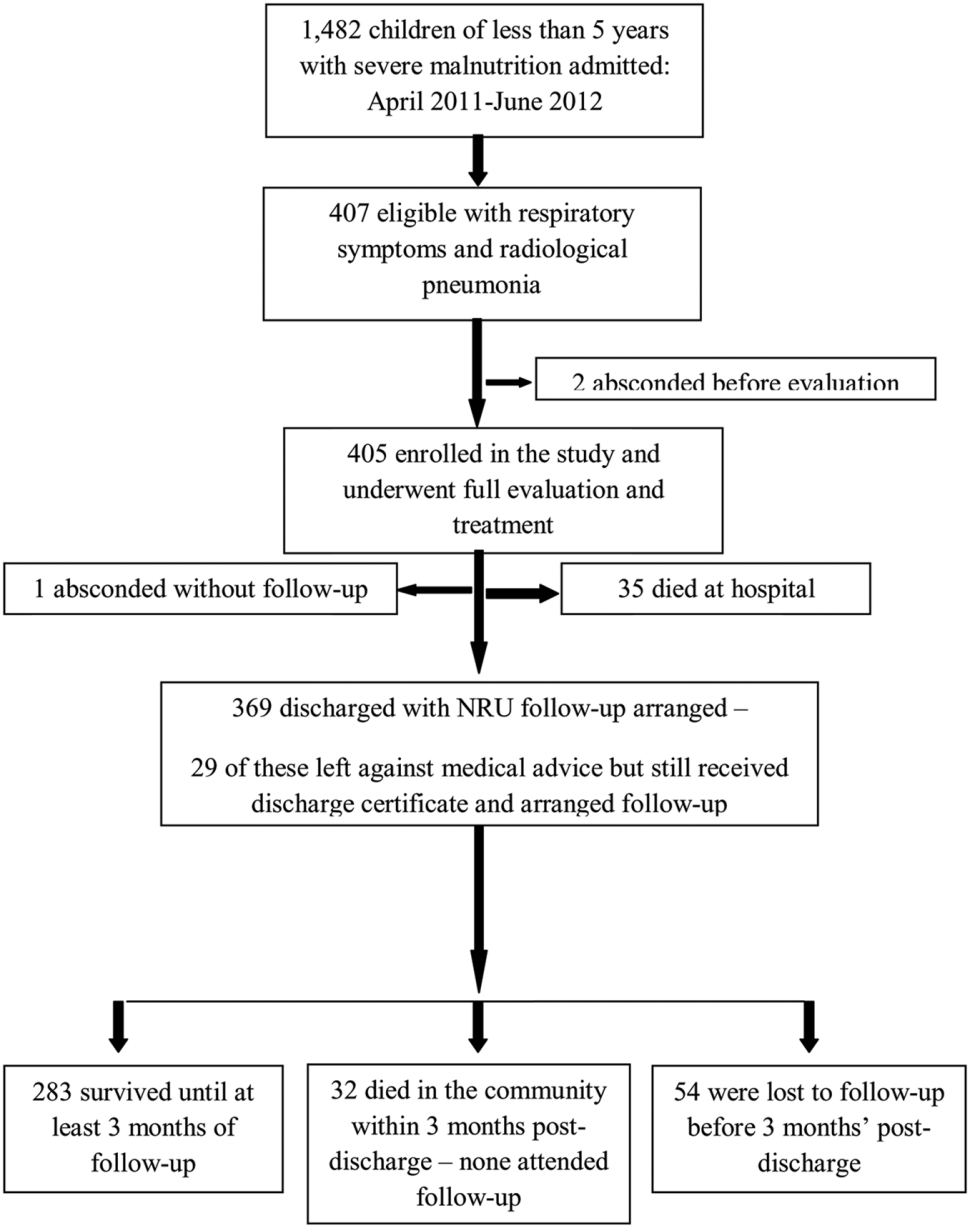
Flowchart of study subjects from admission until final follow-up.

A total of 405 children with severe malnutrition and radiological pneumonia were enrolled at admission and have been described previously [14]. Of these, 35 (8.6%) died as inpatients and 1 patient absconded without follow-up arranged. Figure 1 illustrates a flowchart from admission to follow-up showing that 369 children were discharged with a follow-up plan, including 29 that left against medical advice prior to planned discharge. The median (IQR) age in months of these 369 children was 10 (5, 18) months. The median (IQR) duration of inpatient care before transfer to the NRU for the 340 survivors that were discharged was 6 (4, 10) days, and was of similar duration for the 29 that left against medical advice - 8 (4, 9) days. Of those transferred to the NRU, the majority were discharged early “on risk bond”. Only 43 (13%) remained at the NRU until they reached the standard discharge criteria and median (IQR) duration of hospital stay for these was 18 (12,22) days which compared to only 5 (4,8) days for the remaining 297 that were discharged early.

At 3 months post-discharge, 32 (8.7%) children were known to have died in the community. None of those that died attended any follow-up visit. A further 54 (15%) of the 369 children discharged were lost-to follow-up with no knowledge of outcomes at 3 months post-discharge – Figure 1. Table 1 lists and compares patient characteristics on admission that were associated with known death following discharge and compared to those known to have survived. The features of the 54 children that were lost-to-follow-up but with unknown outcomes are also listed.

**Table 1:** Characteristics of severely malnourished children (<5 years) with pneumonia who died within 3 months following discharge compared to those known to have survived evaluating known risk factors for outcome such as age, severity of malnutrition and severity of pneumonia.

Characteristics	Deaths(N = 32) N (%)	Survivors(N = 283) N (%)	LTFU(n = 54) N (%)	UnadjustedOR (95% CI)
***On admission***				
Age in months: median (IQR)*	6 (3,12)	10 (5, 18)	12 (5, 21)	
Age less than 12 months	23 (72)	157 (56)	27 (50)	2.05 (0.9–4.9)
Male	15 (47)	160 (57)	31 (57)	0.68 (0.3–1.5)
Live outside Dhaka district	10 (31)	60 (21)	9 (17)	1.69 (0.7–4.0)
Poor socio-economiccondition - income <125USD per month	25 (78)	235 (83)	51 (94)	0.73 (0.3–2.0)
History of previouspneumonia prior to present episode*	6 (19)	18 (6)	4 (7)	3.4 (1.1–10.2)
Severe wasting*(z score <−4 weight-for-height/length)	20 (63)	107 (38)	23 (43)	2.74 (1.2–6.2)
Severe underweight*(z score <−5 weight-for-age)	22 (69)	124 (44)	22 (41)	2.82 (1.2–6.7)
Nutritional edema	3 (9)	12 (4)	1 (2)	2.34 (0.5–9.6)
Lower chest wall in-drawing	12 (38)	116 (41)	15 (28)	0.86 (0.4–1.9)
Hypoxemia(arterial oxygen saturation <90% in room air)	3 (9)	22 (8)	2 (4)	1.23 (0.3–4.7)
Confirmed TB	3 (9)	16 (6)	8 (15)	1.74 (0.4–6.9)
Clinical TB – not confirmed	1 (3)	50 (18)	8 (15)	0.15 (0.01–1.1)
***On discharge***				
Duration of hospitalstay in days: median (IQR)	7 (4, 11)	6 (4, 10)	6 (3, 9)	
Left against medical advice*	8 (25)	21 (7)	0 (0)	4.16 (1.5–11.3)
Severe wasting*(z score <−4 weight-for-height/length)	14 (45)	55 (20)	15 (28)	3.40 (1.5–7.8)
Severe underweight*(z score <−5 weight-for-age)	18 (56)	84 (30)	18 (33)	3.05 (1.4–6.8)

*P value significant (<0.05) for comparison of deaths vs. survivors.

LTFU = lost-to follow-up; OR = Odds ratio; CI = Confidence interval; IQR = Interquartile range.

Compared to survivors, those that died post-discharge were younger [median (IQR): 6.5 (3, 12) months], more severely malnourished on admission and more commonly had a previous admission for pneumonia (Table 1). On bivariate analysis, severe wasting on admission (OR 3.64 [95% CI 1.66–7.97], p = 0.001) and age <12 months (OR 2.54 [95% CI 1.1–8.8], p = 0.03) were significantly associated with post-discharge death. Those that died post-discharge were also significantly more malnourished at discharge than those that survived. There was no significant difference in changes to nutritional parameters (by z score) from admission to discharge between those that died and survivors. Post-discharge death was more common in those that were discharged early “on risk bond” from the NRU than those that were discharged once NRU criteria were met with only 1 death in the latter group (P = 0.25).

The median time gap between discharge and death was 22 (9, 35) days. Most deaths occurred within 1 month following discharge and 34% before the first scheduled follow-up visit (Table 2). Characteristics of these early post-discharge deaths such as age and nutritional status were similar to those that died later than two weeks post-discharge (data not shown). There were 86 children discharged receiving anti-tuberculosis treatment [14]. Four of these died within the 3 month follow-up period of which 3 had microbiologically confirmed tuberculosis and the other had clinically diagnosed tuberculosis that was not confirmed microbiologically. Death was recorded more significantly commonly in those that left against medical advice (8 or 28% died of 29) compared to those that were discharged according to usual protocol (24 or 7% of 340) – odds ratio 23.6 (95% CI 9.4–59); P<0.0001.

**Table 2:** Characteristics of post-discharge deaths: timing of death and verbal autopsy findings.

*Time of death after discharge n = 32*		
Timing of death post-discharge	N (%)	Cumulative
Death during weeks 1–2	11 (34%)	34%
Death during weeks 3–4	8 (25%)	59%
Death during month 2	9 (28%)	87%
Death during month 3	4 (13%)	100%
***Caregiver providing verbal autopsy n = 32***		
Mother	18 (56%)	
Father	7 (22%)	
None available*	7 (22%)	
***Verbal autopsy findings n = 25***		
Place of death		
Home	20 (80%)	
At other hospital (local)	4 (16%)	
On the way to hospital	1 (4%)	
**Reported symptoms (new onset)**	**N (%)**	**Duration in days (median, IQR)**
Cough	21 (84%)	5 (3, 7)
Difficulty breathing	18 (72%)	2.5 (2, 4)
Fever	14 (56%)	3.5 (2, 5)
Watery diarrhea	14 (56%)	3 (1.8, 4)
Vomiting	11 (44%)	2 (1, 3)
Abdominal distension	8 (32%)	2 (1.3, 2)
Poor feeding	12 (48%)	3 (2, 5)
White patches on tongue (oral thrush)	4 (16%)	5 (3.5, 6.5)
Treatment		
History of taking treatment during illness	13 (52%)	
Any documentation of treatment	7 (28%)	

*For the 7 deaths without verbal autopsy information, deaths were confirmed by relatives or neighbors when given home address was visited, but the family had moved house and were unable to be contacted or declined further contact with the study team.

Clinical findings from those that had verbal autopsy responses available are shown in Table 2. These findings suggest that deaths usually followed acute lower respiratory tract infections or acute gastroenteritis. Of the 32 deaths, 14 were receiving treatment from a traditional healer compared to only 1 of the 283 survivors at 12 weeks’ post-discharge: unadjusted odds ratio 56.2 (95% CI 15.1–229); P<0.001. Although all the care givers had the access to mobile phone (their own or their relatives), none (0%) of the care givers among the deaths had consulted with study physicians using their mobile phone whereas 115 (41%) of 283 survivors consulted the study physicians by phone (p<0.001).

## Discussion

This study provides original data reporting a high mortality within 3 months of discharge of Bangladeshi children following hospitalization with severe malnutrition and pneumonia. The reported post-discharge mortality of 8.7% is similar to the inpatient mortality for this group, and most of the post-discharge deaths occurred within 1 month following discharge. Previous studies from urban and rural Bangladesh have reported similar or higher post-discharge mortality in children hospitalized with diarrhea, with severe malnutrition recognized as a risk factor for death [9], [10]. Pneumonia is known to be a common co-morbidity in children with severe malnutrition that is associated with inpatient death [4]. We provide original data of post-discharge outcomes in this group.

While studies directly reporting post-discharge mortality of children with severe malnutrition are surprisingly limited [10]–[12], there are data from recent intervention studies in populations of children with severe malnutrition that do follow such children once discharged to the community for home-based management. A recent study that included 393 HIV-uninfected Malawian children with severe malnutrition and with a very low rate of defaulting reported 44 (11%) deaths within 3 months [5]. In another study in Malawi evaluating home-based RUTF, the proportion of those with WHO-defined severe malnutrition that died among the non-intervention group was 6.2% [18]. However, that study population was somewhat different to the one reported here in that it only included children older than 10 months and excluded those who had evidence of systemic infection or severe edema at enrolment.

Admission characteristics that were associated with post-discharge mortality included young age and severity of malnutrition, consistent with previous observations of post-discharge mortality in children [8], [9], [19]. Previous hospitalization for pneumonia and having congenital heart disease were also significantly associated with mortality but the numbers for each of these groups were small. Severity of malnutrition on discharge was associated with poor outcome as was leaving against medical advice. The symptoms present prior to death reported at verbal autopsy suggested recent respiratory or gastrointestinal infection are these symptoms are similar to those reported in previous studies [5], [10], [18].

None of these findings are surprising as young and severely malnourished children are well recognized as being particularly vulnerable to new infections causing severe disease and associated with poor outcomes [8]. Many of these children at discharge were still malnourished and so likely to have persistent and profound immunodeficiency [20]. In addition, they would have frequent exposure to infection, community-acquired upon return to home-based care as well as being at risk from nosocomial infections acquired during the inpatient stay in a crowded hospital.

The study also highlighted potential problems relating to access to care when this particularly vulnerable population developed new symptoms following discharge. Most died at home within one month of discharge with no contact with the hospital staff despite the recent offer of support and advice via mobile phones. It appeared that many sought treatment elsewhere including often with traditional healers. Care seeking with traditional healers has previously been reported as associated with an increased risk of death in children at-risk for severe and rapid-onset disease such as in this population [21], [22].

Our study has a number of important limitations. Primary outcome of survival status at 3 months was not known for 15% of those discharged. The HIV status of the study participants was not known despite this being an important risk factor for a poor outcome in African studies [5]. However, it is unlikely to be an important confounder in this population given that Bangladesh has a very low HIV prevalence among the adult population reported as <0.1% in 2011 [13]. The reported pattern of illness prior to death relied on verbal autopsy which has recognized limitations [23]. Analysis of post-discharge mortality and determinants of risk were not the primary outcomes of this study that determined sample size [14]. Finally, the study does not represent post-discharge mortality among malnourished children that have received complete “nutritional rehabilitation” because most of the study participants were discharged before they met the NRU’s criteria for discharge.

Despite the limitations, this study highlights the potential of a number of interventions that could improve post-discharge mortality in this population. As noted above, most of these children were discharged for home-based care before they met the nutritional criteria set by the NRU. It was reported that the main reason for this was pressure from the family who requested discharge once the child had apparently recovered from pneumonia. However, many of these children were still severely malnourished at discharge and it was observed that this was significantly associated with a poor outcome. Clearly, these children would still be profoundly immunosuppressed and vulnerable to severe infection at the time of discharge. A recent small study from the same institution reported a lower post-discharge death rate (2.8%) at 6-month follow-up among 180 Bangladeshi children with severe malnutrition that had completed full protocol of nutritional rehabilitation [24]. However, the challenge may not be simply to seek to prolong the stay in the NRU until criteria are met as this does have some negative risks as well such as nosocomial infection, overcrowding of the NRU and poor compliance by families. Rather, it may be possible to better select those that would benefit from ongoing care in the NRU, and to improve home-based care to those that are discharged for home-based care.

Two potential options for home-based care that have received recent attention are to provide additional nutritional support as a form of RUTF and/or to provide preventive therapy to reduce the risk of severe infections such as cotrimoxazole preventive therapy (CPT). An intervention study that compared home-based RUTF to standard care in Malawian children with severe malnutrition reported significantly fewer deaths and less relapse among those receiving home-based RUTF at 2 month follow-up [18]. Reported illness of fever, cough and diarrhea were also all significantly less frequent in those that received RUTF. The benefit included those that were still severely malnourished by WHO criteria. CPT is widely recommended for HIV-infected children (and oncology patients) because it improves survival and reduces the incidence of serious infections such as invasive bacterial disease and *Pneumocystis* pneumonia [25], [26]. Future studies, such as recently undertaken in Kenya [27] need to clarify if CPT provides post-discharge benefit for HIV-uninfected children with severe malnutrition. Attempts to influence the gut biome using probiotics have not been beneficial in one recently published study [5]. More intensive follow-up with ready access to health care advice such as by using mobile phones has shown benefit in previous studies [28]. It is likely that different settings may require different solutions given the many potential determinants of mortality in this population. It is also difficult to determine the generalizability of the findings of this study.

In conclusion, post-discharge mortality is common among severely malnourished children with pneumonia and most of these deaths occur within one month of discharge. Risk factors for death include young age and severity of malnutrition and verbal autopsy suggests that death is preceded by recent respiratory or gastrointestinal infection, consistent with limited data from previous studies. There are a number of interventions that could potentially reduce post-discharge mortality in this population but a better understanding of the determinants of poor outcome and evidence from clinical studies are required.

## References

[1] Walker CL, Rudan I, Liu L, Nair H, Theodoratou E, et al (2013) Global burden of childhood pneumonia and diarrhoea. Lancet 381: 1405–1416. 23582727 10.1016/S0140-6736(13)60222-6 PMC7159282

[2] Bejon P, Mohammed S, Mwangi I, Atkinson SH, Osier F, et al (2008) Fraction of all hospital admissions and deaths attributable to malnutrition among children in rural Kenya. Am J Clin Nutr 88: 1626–1631. 19064524 10.3945/ajcn.2008.26510 PMC2635111

[3] Roy SK, Buis M, Weersma R, Khatun W, Chowdhury S, et al (2011) Risk factors of mortality in severely-malnourished children hospitalized with diarrhoea. J Health Popul Nutr 29: 229–235. 21766558 10.3329/jhpn.v29i3.7870 PMC3131123

[4] Chisti MJ, Tebruegge M, La Vincente S, Graham SM, Duke T (2009) Pneumonia in severely malnourished children in developing countries - mortality risk, aetiology and validity of WHO clinical signs: a systematic review. Trop Med Int Health 14: 1173–1189. 19772545 10.1111/j.1365-3156.2009.02364.x

[5] Kerac M, Bunn J, Seal A, Thindwa M, Tomkins A, et al (2009) Probiotics and prebiotics for severe acute malnutrition (PRONUT study): a double-blind efficacy randomised controlled trial in Malawi. Lancet 374: 136–144. 19595348 10.1016/S0140-6736(09)60884-9

[6] Ahmed T, Ali M, Ullah MM, Choudhury IA, Haque ME, et al (1999) Mortality in severely malnourished children with diarrhoea and use of a standardised management protocol. Lancet 353: 1919–1922. 10371570 10.1016/S0140-6736(98)07499-6

[7] Brewster DR, Manary MJ, Graham SM (1997) Case management of kwashiorkor: an intervention project at seven nutrition rehabilitation centres in Malawi. Eur J Clin Nutr 51: 139–147. 9076403 10.1038/sj.ejcn.1600367

[8] Wiens MO, Pawluk S, Kissoon N, Kumbakumba E, Ansermino JM, et al (2013) Pediatric post-discharge mortality in resource poor countries: a systematic review. PLoS One 8: e66698. 23825556 10.1371/journal.pone.0066698 PMC3692523

[9] Roy SK, Chowdhury AK, Rahaman MM (1983) Excess mortality among children discharged from hospital after treatment for diarrhoea in rural Bangladesh. Br Med J (Clin Res Ed) 287: 1097–1099. 10.1136/bmj.287.6399.1097 PMC1549346 6414583

[10] Islam MA, Rahman MM, Mahalanabis D, Rahman AK (1996) Death in a diarrhoeal cohort of infants and young children soon after discharge from hospital: risk factors and causes by verbal autopsy. J Trop Pediatr 42: 342–347. 9009560 10.1093/tropej/42.6.342

[11] Reneman L, Derwig J (1997) Long-term prospects of malnourished children after rehabilitation at the Nutrition Rehabilitation Centre of St Mary’s Hospital, Mumias, Kenya. J Trop Pediatr 43: 293–296. 9364128 10.1093/tropej/43.5.293

[12] Hennart P, Beghin D, Bossuyt M (1987) Long-term follow-up of severe protein-energy malnutrition in Eastern Zaire. J Trop Pediatr 33: 10–12. 3106648 10.1093/tropej/33.1.10

[13] Marais BJ (2010) Does finding M. Tuberculosis in sputum always equal tuberculosis disease? Am J Respir Crit Care Med 181: 195–196; author reply 196–197. 10.1164/ajrccm.181.2.195a 20053971

[14] Chisti MJ, Graham SM, Duke T, Ahmed T, Ashraf H, et al (2014) A Prospective Study of the Prevalence of Tuberculosis and Bacteraemia in Bangladeshi Children with Severe Malnutrition and Pneumonia Including an Evaluation of Xpert MTB/RIF Assay. PLoS One 9: e93776. 24695758 10.1371/journal.pone.0093776 PMC3973596

[15] Cherian T, Mulholland EK, Carlin JB, Ostensen H, Amin R, et al (2005) Standardized interpretation of paediatric chest radiographs for the diagnosis of pneumonia in epidemiological studies. Bull World Health Organ 83: 353–359. 15976876 PMC2626240

[16] World Health Organization (2013) Pocket book for hospital care of children: guidelines for the management of common childhood illnessess Geneva: World Health Organization. 24006557

[17] World Health Organization (2007) International Standard Verbal Autopsy Questionnaire. In The 2007 WHO Verbal Autopsy Instrument. 20–33.

[18] Ciliberto MA, Sandige H, Ndekha MJ, Ashorn P, Briend A, et al (2005) Comparison of home-based therapy with ready-to-use therapeutic food with standard therapy in the treatment of malnourished Malawian children: a controlled, clinical effectiveness trial. Am J Clin Nutr 81: 864–870. 15817865 10.1093/ajcn/81.4.864

[19] Veirum JE, Sodeman M, Biai S, Hedegard K, Aaby P (2007) Increased mortality in the year following discharge from a paediatric ward in Bissau, Guinea-Bissau. Acta Paediatr 96: 1832–1838. 18001338 10.1111/j.1651-2227.2007.00562.x

[20] Waterlow JC, Alleyne GA (1971) Protein malnutrition in children: advances in knowledge in the last ten years. Adv Protein Chem 25: 117–241. 4946702 10.1016/s0065-3233(08)60280-6

[21] Mercer A, Haseen F, Huq NL, Uddin N, Hossain Khan M, et al (2006) Risk factors for neonatal mortality in rural areas of Bangladesh served by a large NGO programme. Health Policy Plan 21: 432–443. 16943220 10.1093/heapol/czl024

[22] D’Souza RM (2003) Role of health-seeking behaviour in child mortality in the slums of Karachi, Pakistan. J Biosoc Sci 35: 131–144. 12537161

[23] Qureshi JS, Samuel JC, Mulima G, Kakoulides S, Cairns B, et al. (2014) Validating a verbal autopsy tool to assess pre-hospital trauma mortality burden in a resource-poor setting. Trop Med Int Health. 10.1111/tmi.12268 24617322

[24] Ashraf H, Alam NH, Chisti MJ, Mahmud SR, Hossain MI, et al (2012) A follow-up experience of 6 months after treatment of children with severe acute malnutrition in Dhaka, Bangladesh. J Trop Pediatr 58: 253–257. 21990106 10.1093/tropej/fmr083

[25] Chintu C, Bhat GJ, Walker AS, Mulenga V, Sinyinza F, et al (2004) Co-trimoxazole as prophylaxis against opportunistic infections in HIV-infected Zambian children (CHAP): a double-blind randomised placebo-controlled trial. Lancet 364: 1865–1871. 15555666 10.1016/S0140-6736(04)17442-4

[26] World Health Organization (2006) Guidelines on cotrimoxazole prohylaxis for HIV-related infections among children, adolescents and adults in resource-limited settings: recommendations for a public health approach. HIV/AIDS Programme, World Health Organization, geneva.

[27] Chang AB, Redding GJ, Everard ML (2008) Chronic wet cough: Protracted bronchitis, chronic suppurative lung disease and bronchiectasis. Pediatr Pulmonol 43: 519–531. 18435475 10.1002/ppul.20821

[28] Zurovac D, Sudoi RK, Akhwale WS, Ndiritu M, Hamer DH, et al (2011) The effect of mobile phone text-message reminders on Kenyan health workers’ adherence to malaria treatment guidelines: a cluster randomised trial. Lancet 378: 795–803. 21820166 10.1016/S0140-6736(11)60783-6 PMC3163847

